# Direct Comparison of Patient-completed and Physician-completed Caprini Scores for Plastic Surgery Patients

**DOI:** 10.1097/GOX.0000000000002363

**Published:** 2019-08-08

**Authors:** Jacob Veith, Willem Collier, W. Bradford Rockwell, Christopher Pannucci

**Affiliations:** From the Division of Plastic Surgery, Department of Surgery, University of Utah School of Medicine, Salt Lake City, Utah

## Abstract

We examined the ability of plastic surgery patients to perform their own venous thromboembolism (VTE) risk stratification using a previously validated patient-completed Caprini risk scoring sheet.

**Methods:**

Patients’ Caprini scores were obtained by an attending physician at an office visit through interview and chart review. Subsequently, patients independently completed a previously validated patient reported scoring sheet. We compared patient and physician reported Caprini scores by each Caprini subquestion and by the overall score. We described discordance of scores using frequencies and proportions, quantified the level of agreement using Cohen’s Kappa coefficient, Spearman’s correlation coefficient, and the Wilcoxon rank-sum test, and visualize scoring differences using Bland–Altman plots, where appropriate.

**Results:**

We prospectively enrolled 50 patients. Only 24% (n = 12) of patients had exact matches in physician-completed and patient-completed Caprini scores. Among the 76% (n = 38) with discrepancies, 26 received a higher patient-reported score (median = 2 points, range 1–8 points) and 12 received a lower patient-reported score (median = −1.5 points, range −1 to −6 points). Existing venous thromboembolism prophylaxis guidelines support chemical prophylaxis for inpatients with Caprini scores ≥7. Among 38 patients with score discrepancies, 8 (21.1%) would have been incorrectly prescribed chemical prophylaxis and 4 (10.5%) would have been incorrectly denied chemical prophylaxis.

**Conclusions:**

Plastic surgery patients cannot reliably calculate their own 2005 Caprini scores. Reliance on patient completed scores alone would promote ~25% of patients receiving inappropriate prophylaxis strategies.

## Abstract 2

Supplemental Digital Content is available in the text.

## INTRODUCTION

The Caprini Risk Assessment Model (RAM) is a validated tool for assessing perioperative risk for venous thromboembolism (VTE), including risk in plastic and reconstructive surgery inpatients.^1,2^ Among plastic surgery patients, the Caprini score identifies extreme variability (15-fold) in postoperative VTE risk, ranging from less than 0.5% to more than 8.5%.^2,3^ Perhaps more importantly, the Caprini score assists plastic surgeons in making treatment decisions about patients who should and should not receive VTE chemical prophylaxis. Specifically, the Caprini score identifies plastic surgery inpatients (Caprini scores of 7–8 and >8) who have a clinically relevant (50% relative reduction, absolute risk reduction of 1.2%–4.5%) and statistically significant decrease in the VTE risk when chemical prophylaxis is provided during the inpatient stay. Based on its clinical impact, major societies such as the American Society of Plastic Surgeons, the American Association of Plastic Surgeons, and the American College of Chest Physicians formally recommend individualized VTE risk stratification using the 2005 Caprini score.^4–6^

Risk scores are designed to optimize medical management by identifying baseline risk and guiding clinicians to make evidence-based decisions about patient care. However, risk scores can be cumbersome and time-consuming. As such, physicians may avoid using them as they are perceived to interfere with efficiency in the physician–patient interactions.^7^ Evaluation of risk, however, is of primary concern in modern medicine and should not be avoided simply because it can be difficult to attain. To remove the burden of risk score completion from the provider, prior studies have examined the utility of patient-completed versions of validated risk assessment tools for issues such as opioid aberrant behavior, varicose vein severity, and fall risk.^8–10^

Caprini et al recently published a version of the 2005 Caprini score designed to be completed by patients, instead of providers. In their initial validation study, the final version of the patient-completed Caprini score was found to have near perfect agreement on all individual questions and the overall score. The first phase of the validation study was conducted in a population of at-risk patients for deep venous thrombosis (DVT), specifically patients recruited from a DVT support group. The latter phases were conducted in a population of inpatients only. Thus, the external validity of the patient-completed form remains in question. This study’s explicit goal was to examine the ability of a general plastic surgery population to reliably and accurately identify their own Caprini risk factors, using a previously validated form.

## METHODS

### Recruitment and Scoring

**Fig. 1.**
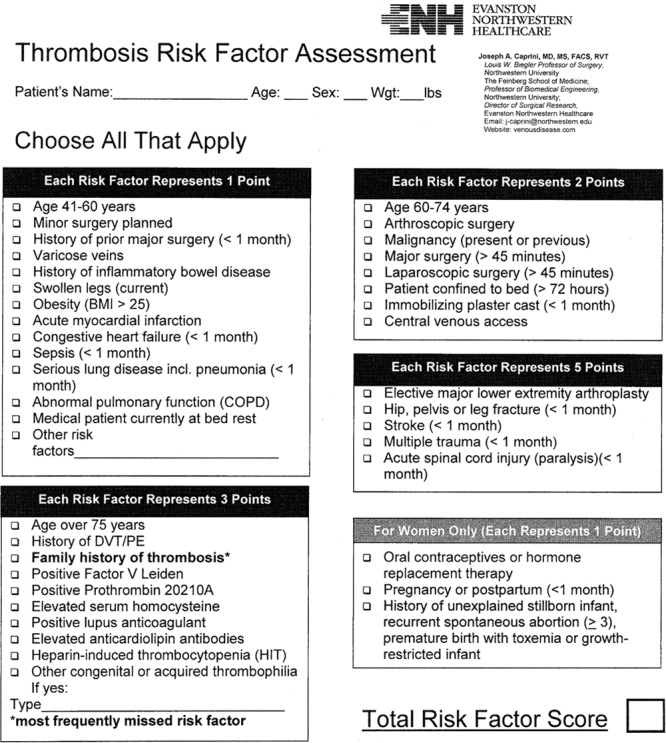
2005 Caprini risk assessment model. COPD, chronic obstructive pulmonary disease.

Our institutional review board reviewed our proposal and approved this study (IRB_0011453, approved 8/15/2018). Plastic surgery patients were prospectively recruited from 2 plastic surgery clinics (those of W.B.R. and C.J.P.) at the University of Utah Hospital and Huntsman Cancer Institute from August 2018 to October 2018. Requirements for inclusion before consent were age greater than 18 years and fluency in English. Patients were informed of the study during their patient visit and were consented for the study after an in-person explanation of the background information, design, and goals of the study. Patients who provided informed consent for participation were asked to fill out a previously validated patient-completed Caprini risk score sheet (See pdf, Supplemental Digital Content 1, which displays previously validated patient-completed Caprini risk score sheet, http://links.lww.com/PRSGO/B169).^11^ Subsequently, their Caprini score based on the 2005 Caprini Risk Assessment Model (Fig. 1)^1^ was obtained by a physician based on an interview and review of the patient’s electronic medical record. The only risk factor left out of the patient-completed score sheet was body mass index, as it had been previously reported that patients inaccurately reported this value.^11^ This risk factor was corrected for as necessary in patient-completed risk scores.

### Statistical Analysis

**Fig. 2.**
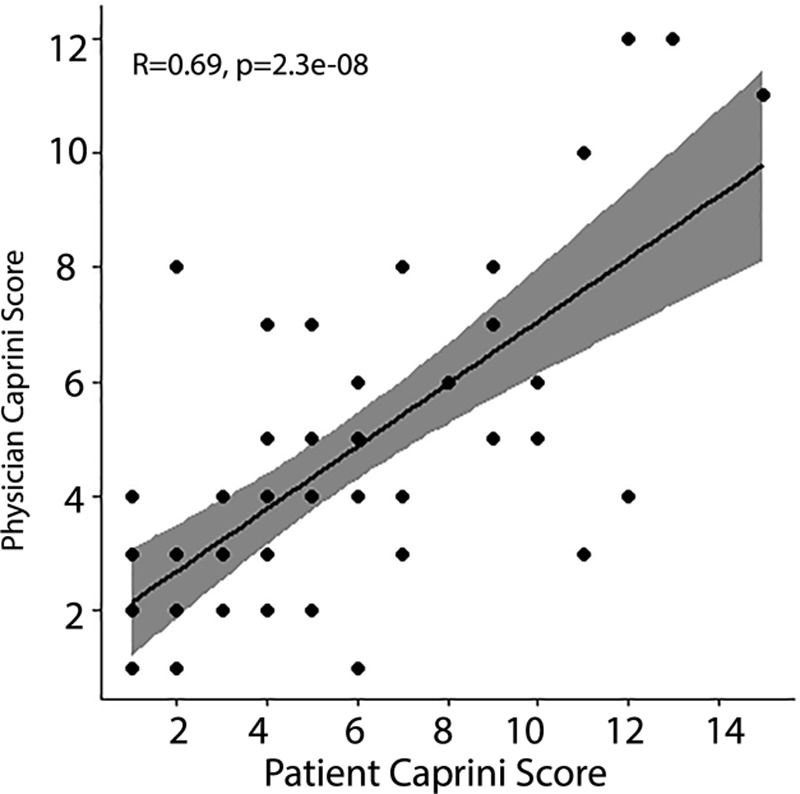
Plot of linear correlation between overall Caprini scores of patients versus physicians.

**Fig. 3.**
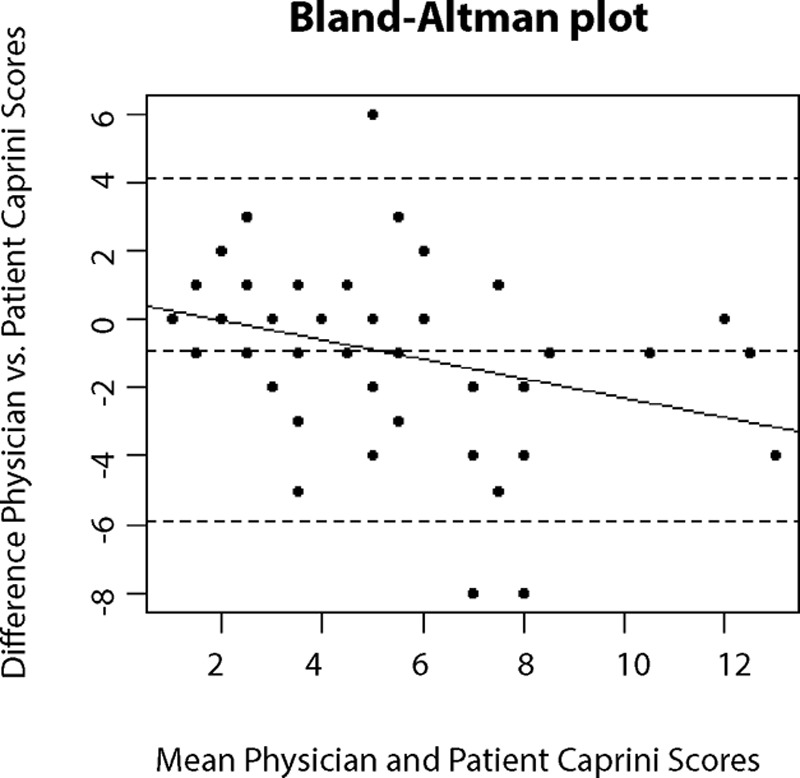
Bland-–Altman plot providing visual evidence of the frequency and extent of disagreement between physician- and patient-reported Caprini scores.

Supplemental Digital Content 1, which displays previously validated patient-completed Caprini risk score sheet, http://links.lww.com/PRSGO/B169, demonstrates that patients answer a total of 29 questions, which combine to create an overall Caprini score. We compared patient versus physician reporting for each of the 29 individual questions and the overall Caprini score. We reported patient–physician discordance by computing the frequency and proportion disagreeing for each of the 29 sub-uestions. We computed Cohens Kappa coefficient and associated *P* value for agreement among the 29 subquestions. Based on the previous analysis of a patient-completed Caprini score, Kappa coefficient of 0.4 or less was considered poor agreement, 0.41–0.60 as moderate agreement, 0.61–0.80 as good agreement, and 0.81–1 as near perfect or perfect agreement.^11^ To compare overall Caprini scores, we compute the Spearman rank correlation coefficient and test against the null hypothesis that this correlation is equal to zero. We additionally compare the distribution of reported Caprini scores across the 2 reporting entities using the Wilcoxon rank-sum test. We assessed the differences between each patient’s own score and physician score to determine how frequently patients with discordant scores would be incorrectly prescribed or incorrectly denied VTE prophylaxis. Finally, we provide Bland–Altman plots and a plot of linear correlation between overall Caprini scores of patients versus physician (Figs. 2 and 3). The Bland–Altman plot provides visual evidence of the frequency and extent of disagreement between physician- and patient-reported Caprini scores, and the relationship between the average rank of the Caprini scores provided and the level of disagreement between reporting entities. Analysis was performed using R-Studio version 1.1.436. All *P* values are associated with 2-sided tests and significance was determined a priori at the 0.05 level.

## RESULTS

There are 29 separate risk factors in the patient-completed Caprini RAM (Table 2). These 29 risk factors align with the 40 factors from the physician-completed Caprini RAM. For the patient completed version, Fuentes et al combined some risk factors from the physician-completed sheet (or excluded in the case of body mass index) to make the sheet more simple for the patient. Of those 29 risk factors, 5 are high risk (worth 5 points toward Caprini score), 10 are moderate risk (worth 2 or 3 points toward Caprini score), and 14 are low risk (worth 1 point toward the Caprini score). Of the 5 high-risk factors, 1 had poor agreement, and 4 could not be assessed. Of the 10 moderate risk factors, 3 had near perfect or perfect agreement, 3 had good agreement, 1 had moderate agreement, and 3 had poor agreement. Of the 15 low-risk factors, 3 had perfect or near perfect agreement, 1 had a good agreement, 4 had moderate agreement, 4 had poor agreement, and 2 could not be assessed.

**Table 1.** Summarizing Patient/Physician Score Discordance across Binary Questions

Risk Factor No.	Risk Factor	Discordance Instances (%)	Cohen’s Kappa Coefficient	*P*
1	41–60 y old*	1(2)	0.957	<0.001
2	61–74 y old**	2(4)	0.901	<0.001
3	>75 y old***	0(0)	1	<0.001
4	>45 min operation within last month*	9(18)	0.618	<0.001
5	Varicose veins within the last month*	1(2)	0.847	<0.001
6	Swollen legs within the last month*	10(20)	0.453	0.001
7	Heart attack within the last month*	0(0)	DNE	DNE
8	Serious infection (pneumonia, cellulitis, sepsis, etc.) within the last month*	3(6)	0.545	<0.001
9	History of inflammatory bowel disease (Crohn’s or ulcerative colitis)*	2(4)	0	DNE
10	History of congestive heart failure*	0(0)	1	<0.001
11	Chronic lung disease not including asthma (COPD, emphysema, etc.)*	2(4)	0.485	<0.001
12	Currently on hormonal birth control or hormone replacement therapy	2(4)	0	DNE
13	Pregnant or had a baby within last month*	0(0)	DNE	DNE
14	History of an unexplained stillborn, more than 3 spontaneous abortions, premature birth with preeclampsia, or child with inappropriately low birthweight*	2(4)	0.479	0.001
15	History of cancer, leukemia, or malignancy (past or current)**	9(18)	0.589	<0.001
16	Nonremovable leg cast within last month**	1(2)	0.658	<0.001
17	PICC line, central venous access catheter, or port within last month**	1(2)	0.878	<0.001
18	History of blood clot***	3(6)	0.806	<0.001
19	Family history of blood clot***	3(6)	0.789	<0.001
20	Abnormal blood test with increased risk for clotting (clotting disorder)***	4(8)	0	DNE
21	Currently bedrest (<3 d)*	10(20)	0.135	0.057
22	Chronic bedrest (≥3 d)**	4(8)	0.31	0.002
23	Hip or knee replacement surgery within last month*****	0(0)	DNE	DNE
24	Broken hip, pelvis, or leg within last month*****	0(0)	DNE	DNE
25	Serious trauma or polytrauma within last month*****	2(4)	0	DNE
26	Spinal cord injury resulting in paralysis within last month*****	0(0)	DNE	DNE
27	Stroke or transient ischemic attack within last month*****	0(0)	DNE	DNE
28	Scheduled minor surgery (<45 minutes) within next month*	3(6)	0	DNE
29	Scheduled major surgery (>45 minutes) within next month **	11(22)	0.048	0.704

If DNE is in both the coefficient and *P* value columns, the risk factor was never chosen by either patient or physician. If DNE is in only the *P* value column, the value was chosen at least once by either the patient or physician and never chosen by the other. Number of * = Points towards Caprini score.

COPD, chronic obstructive pulmonary disease; DNE, did not evaluate; PICC, peripherally inserted central catheter.

**Table 2.** Summarizing Concordance/Discordance in Overall and Categorized Patient versus Physician Caprini Scores

Entity Reporting	Median (IQR) Caprini Scores	SPR	*P* (Null: SPR = 0)	P (Rank-sum Test)
Patient	5(3,7)	0.65	<0.001	0.251
Physician	5(2,6)			

IQR, interquartile range; SPR, Spearman Rank-correlation.

A total of 50 patients were prospectively enrolled in the study. Of these patients, 29 (56%) were male and average age was 49.5 years. Comparing the patient- and physician-completed scores, Spearman’s correlation was 0.694, with corresponding *P* < 0.001 for the overall Caprini score, indicating we reject the null hypothesis of zero correlation at any reasonable significance level. This correlation can be visualized in Figure 2. The results of the Wilcoxon rank-sum test (*P* = 0.251) has similar implications in that we fail to reject the null hypothesis that the probability of a randomly selected patient reported score being higher than that of a physician reported score is equivalent to it being lower.

**Fig. 4.**
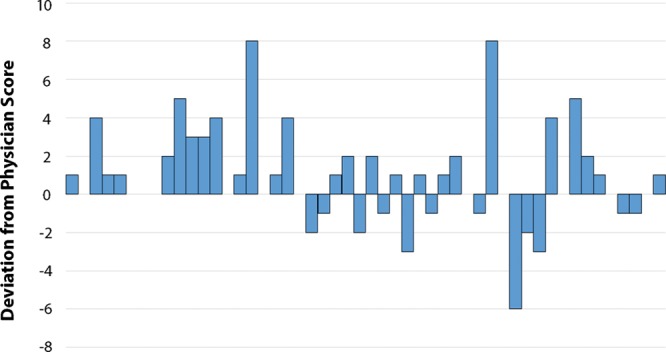
Patient-completed Caprini score deviation from physician-completed score.

Despite that these statistical tests indicate that patient- and physician-reported scores are correlated and similarly distributed, of the 50 patients, only 12 (24%) had exact matches in physician-completed and patient-completed risk scores. There were 26 (42%) total patients with higher patient-reported scores and 12 (24%) with higher physician-reported scores. Figure 4 demonstrates the deviation of patient completed scores form the physician’s score. Of the patients with higher patient-reported scores, the difference ranged from 1 to 8 points higher than their physician-completed score and the median difference was 2 points greater. Of the patients with lower patient-reported scores, the difference ranged from 1 to 6 points lower than their physician-completed score and the median difference was 1.5 points fewer. The Bland–Altman plot (Fig. 3) provides additional means to visualize discordance between patient and physician reported Caprini scores. Each data point indicates the difference of the physician- and patient-reported scores via the vertical axis, and the average of the 2 scores for each subject via the horizontal axis. The 2 horizontal lines on the extreme ends of the plot indicate where 95% of the data fall, which in this case indicates that it is not highly unreasonable for an overall patient-reported Caprini score to be nearly 6 points higher than that of the physician. One may also notice the downward trend line, which indicates that, as the average of the 2 Caprini scores increases, it is more likely that the patient is reporting a higher Caprini score than their physician.

A total of 66% (n = 38) of patients did not have scores that correlated with that of the physician. More importantly, 24% (n = 12) would have been incorrectly categorized. Eight patients would have been incorrectly prescribed chemical prophylaxis and 4 patients would have been incorrectly denied chemical prophylaxis.

## DISCUSSION

Patient-reported Caprini scores had a good (though not “near perfect” or “perfect”) correlation with physician-completed risk scores. Statistical tests also indicated that the distribution of such scores did not differ considerably across reporting entities. However, our data clearly demonstrate that utilization of patient-reported Caprini scores to guide chemical prophylaxis provision would provide inappropriate prophylaxis (either inappropriately provide or inappropriately withhold) in one in 4 patients. These situations could lead to unnecessary risk of VTE or bleeding. Based on this data, we cannot recommend utilization of patient completed Caprini scores to guide VTE prophylaxis strategies after plastic surgery patients’ procedures. Our data show that ~50% of risk factors had unacceptable agreement between patients and providers. Existing data from the VTEPS study, in addition to guidelines from the ASPS VTE task force,^3,5^ support provision of chemical prophylaxis to plastic surgery inpatients with Caprini scores ≥7. However, we also note that as the average reported Caprini scores increase, more patients tend to report higher scores than their physician. As such, patient-reported Caprini scores ≥7 should be treated with caution.

We examined each of the risk factors that were found to have poor or moderate concordance. Patients may not define certain risk factors in the same way as physicians. For instance, “serious trauma,” “abnormal blood test,” “serious infection,” and “swollen legs” all were chosen unnecessarily by the patient. Bedrest status and obstetric risk factors appeared to confuse the patients as well. Patients also showed confusion in operative plan, especially with regards to length of the planned surgical procedure. “Scheduled major surgery (>45 min)” and “scheduled minor surgery (<45 min)” both had poor agreement. These risk factors may need to be refined on a future version of this patient-completed RAM.

Fuentes et al^11^ had 9 risk factors that did not have “perfect” agreement: recent major surgery, recent serious infection, history of cancer/malignancy, recent central line access, family history of blood clot, current bedrest, recent bedrest, scheduled major surgery, and scheduled minor surgery. However, all of these risk factors had “near perfect” agreement. Although our results do not closely resemble each other in correlation, we did find that some of these factors had lower agreement. It is clear that patients do not always understand their surgical plan, what bedridden status entails as defined by the physician, or what qualifies as a serious infection.

Despite promising results in preliminary studies, patient-reported risk scores should be used with caution. Relevant patient populations should be used to assess external validity of any self-reported risk stratification tools. Webster and Webster^12^ created the Opioid Risk Tool and showed a high degree of sensitivity and specificity in predicting aberrant behaviors in opioid-prescribed patients. However, a reassessment by Clark et al^13^ showed the tool to be no better chance at predicting opioid aberrant behavior. Although self-reported risk scores are attractive clinical tools, they should not be used to completely replace the work up of a qualified clinician. Pietz and Peterson^14^ compared self-reported health status tool to the diagnostic work up of a physician; they suggest that self-reported risk scores may contain unique information and should be used in conjunction with the physician’s assessment based on interview, exam, and diagnostic work-up.

This study has several limitations worth noting. Some risk factors from the Caprini RAM occur infrequently in plastic surgery patients. These include genetic hypercoagulability, recent hip/knee replacement surgery, recent broken hip/pelvis/leg, recent stroke/transient ischemic attack, recent heart attack, current/recent pregnancy, or recent spinal cord injury with paralysis. Despite enrolling 50 patients, we were unable to rigorously examine these factors. However, we believe that our data from more common risk factors are probably generalizable. Sometimes, it is unclear from their charted history why a patient chose a risk factor and they cannot be reached for an explanation that may be helpful. Use of the electronic medical record for the physician-obtained score could lead to confounding errors if patients have ever provided incorrect information on their medical history, although we tried to avoid this type of error by only using the electronic medical record when information was missed during the visit and mainly using previous clinic visit notes from the attending physicians who participated in this study. Another limitation is our choice to only validate in English, as the assessed tool has been validated in English, Spanish, Arabic, and Polish.^15^ Finally, we chose provider-completed scores as the gold standard for Caprini score (eg, this was the standard for comparison). However, as the study’s principal investigator has completed thousands of Caprini scores,^2,3,6,16–19^ we consider this to be a valid choice.

## CONCLUSIONS

It is important to engage patients in their own care, but it is ultimately the responsibility of the physician to ensure that safe and appropriate decisions are made regarding patient care. Patient-reported risk scores should be used with caution. We found that plastic surgery patients cannot reliably calculate their own Caprini scores using an established patient-centric tool. Blind reliance on patient-reported Caprini scores would facilitate about one plastic surgery patient in 4 receiving an inappropriate chemical prophylaxis regimen (either inappropriately providing or withholding chemical prophylaxis). Future studies could modify the existing internally validated form for plastic surgery patients, and a plastic surgery-centric form could be validated.
